# Efficacy and resistance in de novo combination lamivudine and adefovir dipivoxil therapy versus entecavir monotherapy for the treatment-naive patients with chronic hepatitis B: a meta-analysis

**DOI:** 10.1186/1743-422X-11-59

**Published:** 2014-03-28

**Authors:** Fen Liu, Xiwei Wang, Fang Wei, Huaidong Hu, Dazhi Zhang, Peng Hu, Hong Ren

**Affiliations:** 1 Department of Infectious Diseases, The Second Affiliated Hospital of Chongqing Medical University, Chongqing, China; 2 Department of Infectious Diseases, Institute for Viral Hepatitis, Key Laboratory of Molecular Biology for Infectious Diseases, Ministry of Education, The Second Affiliated Hospital of Chongqing Medical University, Chongqing, China

**Keywords:** Adefovir dipivoxil, Combination, De novo, Entecavir, Lamivudine, Naive

## Abstract

**Background:**

Currently, there is no consensus on the efficacy and resistance of de novo combination therapy versus monotherapy for treatment naive patients of chronic hepatitis B (CHB).

**Objectives:**

The aim of this study was to evaluate the effectiveness and resistance of de novo combination of lamivudine (LAM) and adefovir dipivoxil (ADV) compared with entecavir (ETV) monotherapy for nucleos(t)ide–naive patients with CHB.

**Study design:**

Publications on the effectiveness and resistance of LAM plus ADV versus ETV monotherapy for nucleos(t)ide-naive patients with CHB were identified by a search of PubMed, Embase, the Cochrane Library, Web of science, OVID, and CBM (Chinese Biological Medical Literature) until May 1, 2013. Biochemical response, hepatitis B e antigen seroconversion, and viroligic response were extracted and combined to obtain an integrated result. Viral resistance and safety were reviewed.

**Results:**

Five eligible studies (328 patients in total) were included in the analysis. LAM plus ADV combination therapy produced more rapid HBV DNA reduction rate at 12 weeks than that of ETV monotherapy. At 48 weeks, the combination group had superior viroligic response rates compared with ETV group (90.0% vs. 78.9%, P=0.01). The difference in the ALT normalization and HBeAg seroconversion rates was not found. At week 96, LAM + ADV was more effective than ETV in ALT normalization [RR = 1. 11, 95% CI (1.02, 1.21), P =0.01] and HBeAg seroconversion [RR = 2.00, 95% CI (1.26, 3.18, P=0.003)], and no significant difference was found in the virologic response (P =0.23). No viral resistance occurred in combination therapy and six patients in ETV group were experienced with viral breakthrough. Both groups were well tolerated.

**Conclusion:**

The de novo LAM plus ADV combination therapy for treatment-naïve patients with CHB was greater than ETV monotherapy in both biochemical response and HBeAg seroconversion rate up to 96 weeks. The rate of emergence of viral resistance in the combination group was less than that in the ETV monotherapy.

## Introduction

Nucleos(t)ide analogs(NAS)have become the mainstay of CHB treatment mainly due to their profound viral suppressive effects, the convenience of single daily dosing and relative lack of significant side effects. A major shortcoming of NAS is the high rate of virologic relapse when treatment is discontinued [1]. Therefore, treatment must often be administered long-term. Unfortunately, prolonged therapy is associated with the development of drug resistance [2]. Available clinical data has shown that the emergence of drug-resistant can not only compromise the initial clinical benefits, but also lead to hepatitis flares, hepatic decompensation and even death [3,4]. Thus, the prudent selection of appropriate agents for the initial treatment of CHB patients to achieve an efficacy, while simultaneously avoiding the emergence of resistance, is a vital clinical concern.

Currently, there are five NAS that have been approved to treat CHB. LAM was the first one to be approved by the United States Food and Drug Administration and has a well-established safety and efficacy profile. However, it has a high incidence of drug-resistance increasing from 24% in 1 year to approximately 70% in 5 years [5,6]. ADV has an efficacy comparable to that of LAM, but with a low drug resistance rate, and no cross resistance with other nucleoside analogues. Telbivudine (LDT) has a potent efficacy and a relatively high seroconversion rate. ETV, known for its potent antiviral effects, has a high genetic barrier to resistance, as more than three sites are required for drug resistance to develop [7]. ETV also has been recommended as a first-line therapy agent for the naive patients with CHB in the updated Asian-Pacific consensus statement on the management of CHB [8]. However, the rates of HBeAg loss and seroconversion are very low with ETV [9,10]. Tenofovir disoproxil fumarate (TDF) is also been recommended as the first-line therapy agent. However, in some countries, it is not yet widely available or used. LAM was selected for study due to the abundant clinical experience and lowest cost. Evidence-based medicine identified that combination therapy could reduce drug-associated resistance to ensure long-term therapy [11], especially, for LAM resistant and liver transplanted patients [12,13]. Results of available studies have demonstrated that add-on ADV for LAM-resistant patients enhances the viroligical and biochemical responses [14], and the combination of ADV and LAM results in more effective in viral suppression and less risk of developing genotypic resistance, compared with ADV monotherapy [15,16]. Unfortunately, a substantial proportion of patients treated with LAM-plus-ADV combination therapy show a suboptimal virologic response, and some even develop multidrug resistance [17-19]. On the other hand, some studies reported that not only did the de novo LAM plus ADV combination reduce HBV-DNA detectability, enhance ALT normalization and HBeAg seroconversion, but no cases of genotypic resistance had occurred [20]. Therefore, LAM and ADV were selected for study of de novo combination treatment. The purpose of the study described here was to systematically review and meta-analyze all published studies designed to treat CHB naive patients with LAM plus ADV therapy and compare it with ETV monotherapy in terms of efficacy and viral resistance.

## Methods

### Literature search

We searched the following databases: PubMed, Embase, Web of Science, The Cochrane Library, OVID, and CBM (Chinese Biological Medical Literature) until May 1, 2013. Of these databases, CBM database provides literature in Chinese. The search process was designed to find all studies involving terms: “chronic Hepatitis B”, “entecavir”, “adefovir dipivoxil”, “lamivudine” (and multiple synonyms for each term). Reference lists from retrieved documents were also searched. Computer searches were supplemented with manual search. Two authors (Fen Liu and Xiwei Wang) independently screened all citations and abstracts to identify potentially eligible studies. Discrepancies were resolved with the assistance of an arbiter (Peng Hu) when necessary.

### Inclusion and exclusion criteria

The following inclusion criteria were included: (1) randomized controls, prospective case-controls, cohort study designs. (2) HBsAg positive for at least 6 months prior to enrollment regardless of hepatitis B e antigen (HBeAg) status. (3) All patients had never received antiviral treatment previously. (4) Intervention: studies directly comparing LAM 100 mg/d plus ADV 10 mg/d and ETV 0.5 mg/d, with a duration more than or equal to 24 weeks. (5) All patients had to have excellent compliance in taking the antiviral agents. Our search was limited to human studies and the following exclusion criteria were used: (1) non-comparative study; retrospective study, observational study. (2) Insufficient analytical information regarding treatment schedule, follow-up. (3) Patients co-infected with hepatitis A, C, D and E virus, who had decompensated liver disease, hepatocellular carcinoma (HCC). (4) Prior liver transplantation and concomitant renal failure.

### Efficacy measures

The rates of biochemical response, virologic response, and HBeAg seroconversion were used as primary efficacy measures. Emergence of viral resistance and the safety profiles were used as secondary efficacy measures. Biochemical response was defined as normalization of ALT levels. Virologic response was defined as attainment of undetectable levels of HBV DNA (<1 × 10^3^ copies/mL). HBeAg seroconversion was defined as HBeAg disappearance and HBeAg antibody appearance. Viral breakthrough was defined as an increase in serum HBV DNA by 1 log_10_ copies /mL above a nadir on two or more consecutive occasions at least 1 month apart. LAM-, ADV-, ETV-associated mutations were detected by directing sequencing for patients with viral breakthrough. The safety was assessed by compiling adverse events including renal dysfunction, decompensation of cirrhosis and HCC.

### Data extraction

Two investigators independently screened abstracts, selected the studies and performed the data extraction. The conflict in data extraction was resolved by discussion among investigators and reference to the original articles. When several publications pertaining to a single study were identified, the most recent and complete publication was used to prevent duplication of data.

### Quality assessment

Quality of included study was assessed based on following criteria: (1) For RCT: methodological quality was assessed using the Jadad quality scale. We examined the allocation sequence generation, allocation concealment, application of blinding method, and dropouts and withdrawals. (2) For cohorts, the quality of studies was assessed by the Newcastle-Ottawa Scale (NOS) based on the following indicators: selection of cohorts, comparability of cohorts and assessment of the outcomes. Discrepancies were resolved with the assistance of an arbiter (Peng Hu) when necessary.

### Study quality

One study was an RCT and stated the method of randomization, withdrawal and allocation concealment, but did not describe the blinding. Accordingly, it received a Jadad score of 4. The other reports were on cohort studies with defined inclusion and exclusion criteria and definitions of the treatment responses. All study populations had comparable baseline characteristics between the LAM + ADV and ETV groups. However, one study did not follow up long enough for outcomes to occur, so it received a score of 8. The others had scores of 9. Discrepancies were resolved with the assistance of an arbiter (Peng Hu) when necessary.

### Statistical analysis

Data analysis was carried out with the use of Review Manager Software 5.0 (Cochrane Collaboration, Oxford, United Kingdom). Outcomes were analyzed on an intent-to-treat basis. For each eligible study, dichotomous data were presented as relative risk (RR) with 95% confidence intervals (CI). Statistical heterogeneity was evaluated by the chi-square and I-square (I^2^) tests, with significance set at P < 0.10. In the absence of statistically significant heterogeneity, the fixed-effect method was used to combine the results. When heterogeneity was confirmed, the random-effect method was used. Additionally, sensitivity analysis was carried out if low quality studies were included. The overall effect was tested using z scores, with significance set at P < 0.05.

## Results

**Figure 1:**
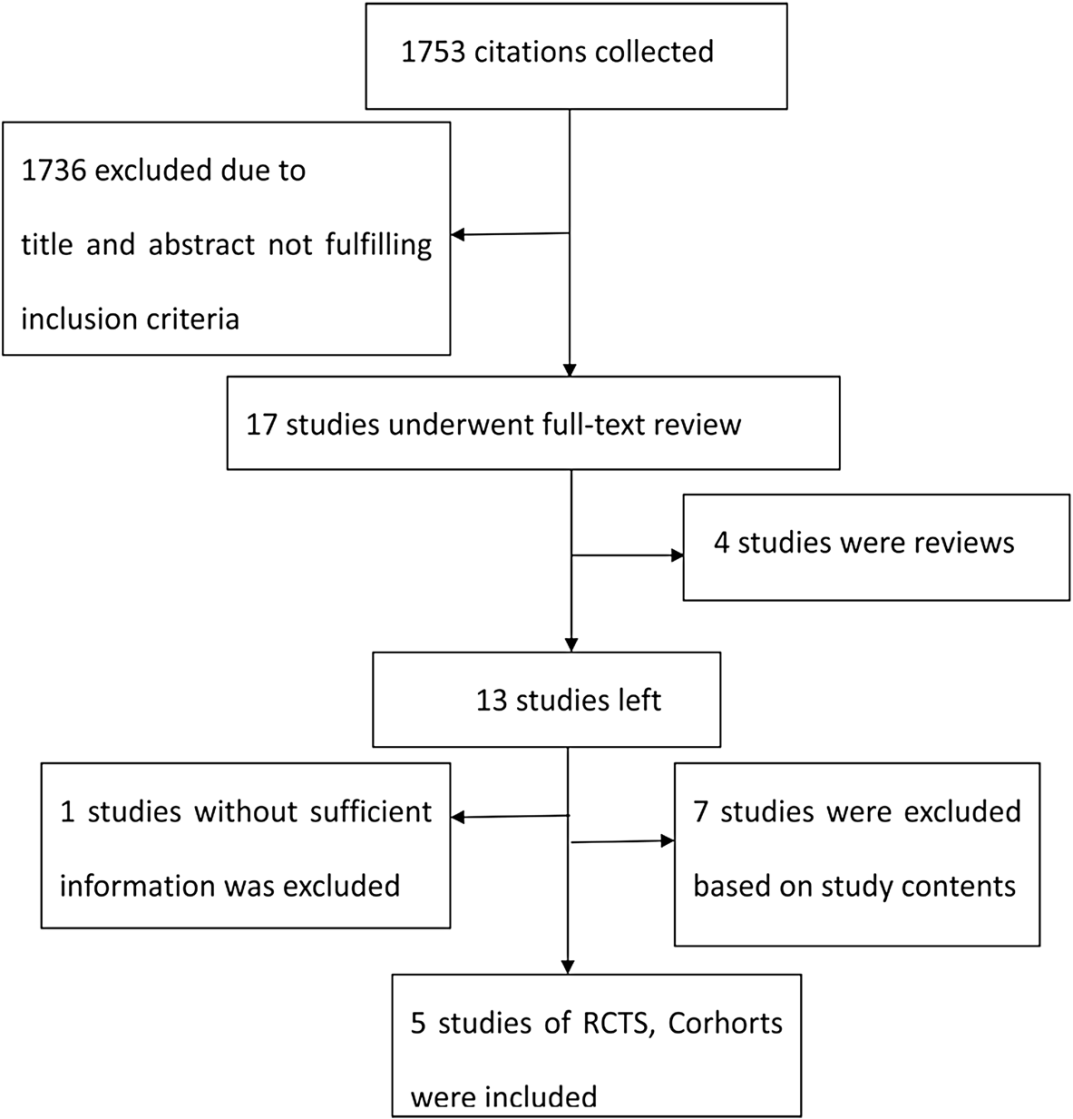
Results of literature search.

We initially identified 1753 potentially eligible citations. 1739 redundant publications, reviews, and meta-analysis were excluded. After referring to full text, seven studies did not satisfy the inclusion criteria and were removed from consideration. Two studies were presented in abstract form. One was found with the full-text which had been included in the study, and the other was excluded because of a lack of sufficient statistical data. The remaining five studies [21-25] contained 328 patients in total, of whom 161 were included in the LAM plus ADV combination group, and 167 were included in the ETV group (Figure 1). Of the five studies, two were published in English, and the others were published in Chinese. All studied populations had comparable baseline characteristics between the two groups. Detailed information was summarized in Table 1. 

**Table 1:** Characteristic of the included studies in this meta-analysis

**Study**	**Location**	**Study design**	**Sample size (n)**		**Sex (M/F) (n)**		**Age (yrs)**		**HBeAg (+/-) (n)**		**Regimen**		**Therapy period**	**Baseline ALT (U/L)**		**HBV DNA level (Copies/mL)**		**Detection limit of HBV DNA**
			**LAM + ADV**	**ETV**	**LAM + ADV**	**ETV**	**LAM + ADV**	**ETV**	**LAM + ADV**	**ETV**	**LAM + ADV**	**ETV**	**LAM + ADV**	**LAM + ADV**	**ETV**	**LAM + ADV**	**ETV**	
Yu [22]	China	Cohort	54	50	47/7	44/6	35.8 ± 8.6	37.3 ± 9.5	36/18	36/14	LAM 100 mg/d + ADV 10 mg/d	0.5 mg/d	96 week	175.2 ± 123.0	144.5 ± 106.8	6.2 ± 1.3㏒10	6.0 ± 1.7㏒10	<300 Copies/mL
Wang [21]	China	Cohort	31	40	28/3	34/6	31 ± 6.78	29.8 ± 6.0	11/20	15/25	LAM 100 mg/d + ADV 10 mg/d	0.5 mg/d	48 week	165.58 ± 80.58	140.68 ± 67.68	1.42*10^6	9.04*10^5	<10^3 Copies/mL
Wei [25]	China	Cohort	20	22	14/6	14/8	35 ± 6.89	37 ± 6.75	20/0	22/0	LAM 100 mg/d + ADV 10 mg/d	0.5 mg/d	104 week	NA	NA	NA	NA	5*10^2 Copies/mL
Zhang [24]	China	RCTS	35	35	NA	NA	43 ± 9	43 ± 9	+:35%	+:23%	LAM 100 mg/d + ADV 10 mg/d	0.5 mg/d	96 week	242 ± 112	249 ± 100	8.0 ± 0.6㏒10	8.1 ± 0.6㏒10	<10^3 Copies/mL
Jayakumar [23]	India	Cohort	21	20	M:19%	M:16%	38.86 ± 12.08	42.15 ± 17.11	10/11	15/5	LAM 100 mg/d + ADV 10 mg/d	0.5 mg/d	24 week	53 (29–163)	44 (17–151)	5.71 (4.2-9.5)㏒10	7.69 (4.0-8.5)㏒10	<400 Copies/mL

NA: not available; LAM: lamivudine; ADV: adefovir; ETV: entecavir; RCTs: randomized controlled trials

### Virologic response

**Figure 2:**
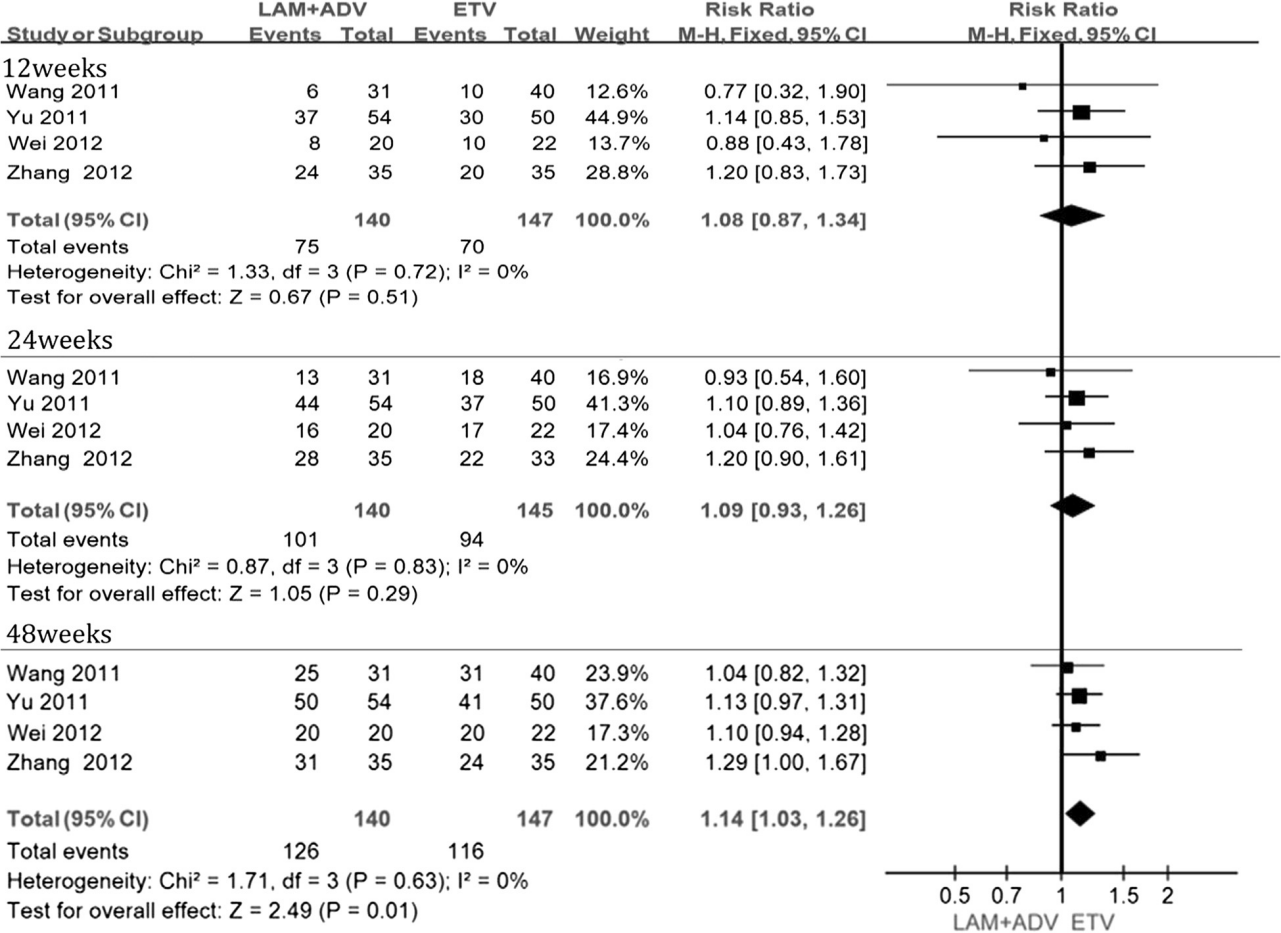
Effect of LAM + ADV vs. ETV on virologic response.

Four studies [21,22,24,25] reported virologic response rates after 12, 24, and 48 weeks. According to chi- and I square (I^2^), heterogeneity revealed no significant differences between treatment groups. The fixed-effect approach was used to estimate of the relative risk of LAM + ADV versus ETV monotherapy. The results showed that the virologic response rates were obviously higher in the combination group than that of ETV monotherapy (53.6%, 72.1%, 90.0% vs. 47.6%, 64.8%, 78.9% at 12, 24, and 48 weeks, respectively). No significant heterogeneity was found at virologic response between two groups at 12, and 24 weeks (P =0.51, P =0.29). However, at week 48, the differences in virologic response rates were statistically significant [RR = 1. 14, 95%CI (1.03, 1.26), P =0.01] (Figure 2, Table 2).

**Table 2:** Virologic response results

**Follow up time(weeks)**	**Number of studies**	**Test of hetergeneity**		**Analysis model**	**Results**		**RR (95% CI)**	**P-value**
		**I**^**2**^	**P**		**LAM + ADV (n/N)**	**ETV (n/N)**		
12	4	0%	0.72	Fixed effect model	75/140	70/147	1.08[0.87,1.34]	0.51
24	4	0%	0.83	Fixed effect model	101/140	94/145	1.09[0.93,1.26]	0.29
48	4	0%	0.63	Fixed effect model	126/140	116/147	1.14[1.03,1.26]	0.01
96	3	82%	0.003	Randomed effect model	102/106	87/105	1.13[0.93,1.38]	0.23

**Figure 3:**
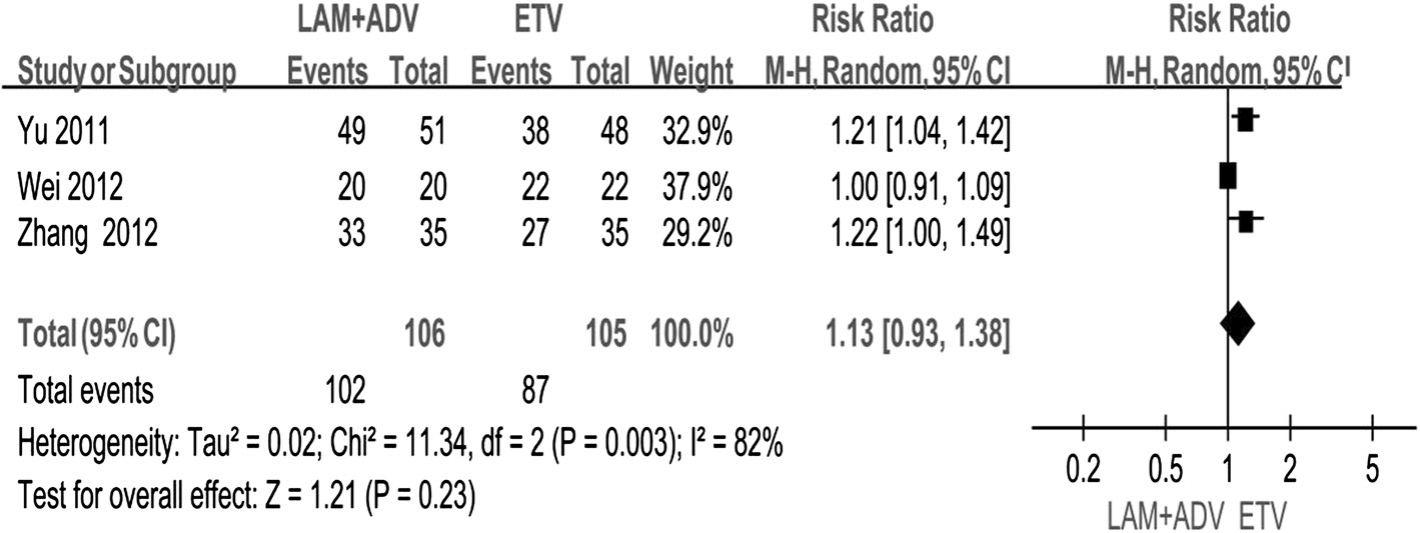
Effect of LAM + ADV vs. ETV at 96 weeks on virologic response.

Only three studies [22,24,25] reported virologic responses at 96 weeks. Chi- and I square (I^2^) analyses identified significant heterogeneity in virologic responses between the two groups [Tau^2^ = 002, Chi^2^ = 11.34, df = 2 (P = 0.003), I^2^ = 82%]. Therefore, a random-effect approach was used which indicated that the virologic response was higher in the combination therapy group than that in the ETV monotherapy group (96.2% vs. 82.8%). However, no significant differences were found [RR = 1. 93, 95%, CI (0.93, 1.38), P =0.23] (Figure 3).

### Biochemical responses

**Figure 4:**
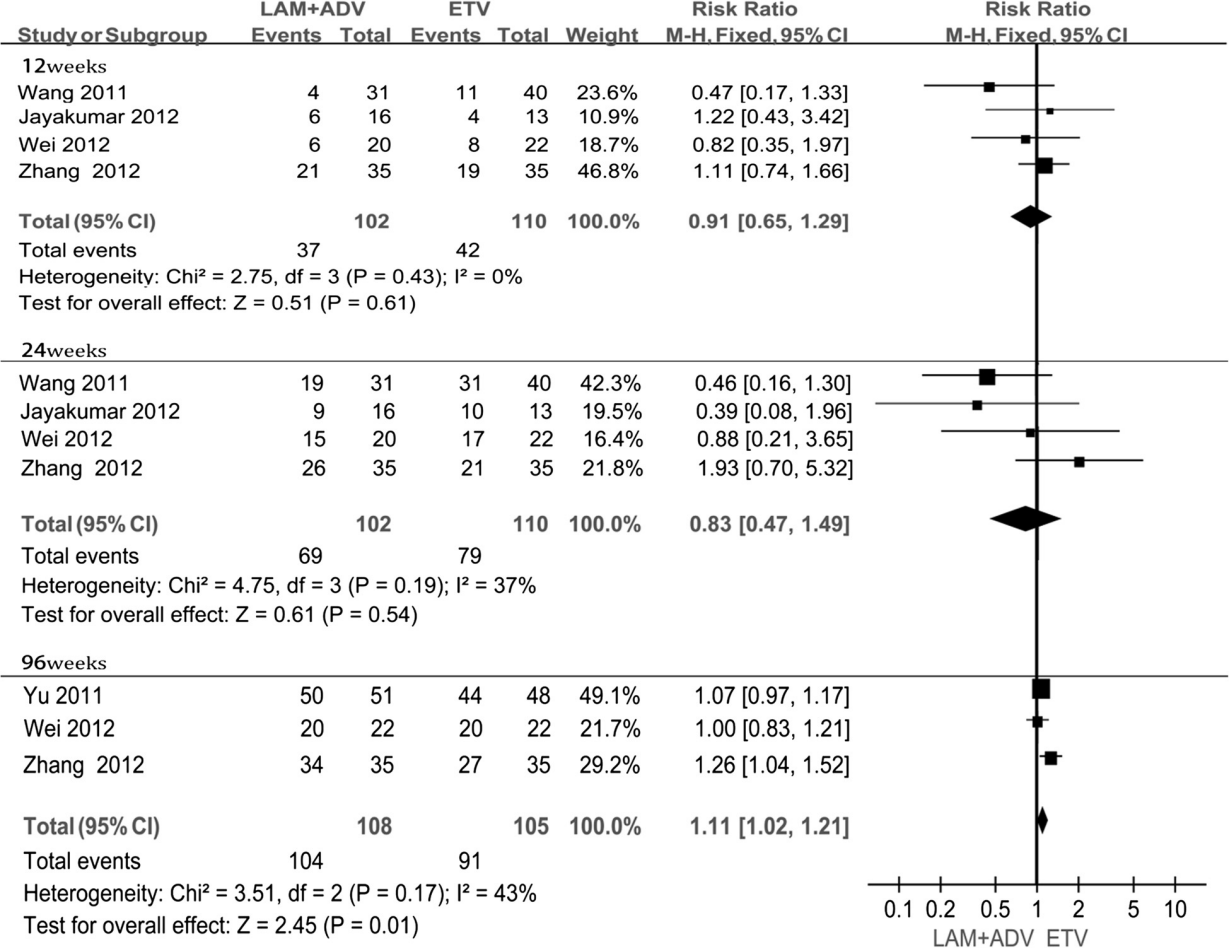
Effect of LAM + ADV vs. ETV on biochemical response.

Four studies [21,23,25] showed the biochemical response rates at weeks 12, and 24. Chi-and I square (I^2^) analyses showed no significant heterogeneity between treatment groups [Chi^2^ = 2.75, df = 3 (P = 0.43); I^2^ = 0%]; [Chi^2^ = 4.75, df = 3(P = 0.19; I^2^ = 37%)]. The fixed-effect approach was used. Another four studies [21,22,24,25] provided the rates of ALT normalization at 48 weeks treatment. Heterogeneity was found between these studies [Tau^2^ = 0.01, Chi2 = 9.31, df = 3, (P = 0.03), I^2^ = 68%]. Thus, a random-effects model was used. There were no statistical significant differences between groups in terms of the ALT normalization rates at 12, 24, and 48 weeks after treatment ( P =0.61, P =0.54 , P =0.21, respectively), although the proportion in the combination group was lower than that of in the ETV monotherapy group after 12, 24 weeks post treatment (36.3% vs. 38.2%, and 67.6% vs. 71.8%, respectively), and was higher than that obtained in the monotherapy group at 48 weeks (91.4% vs. 81.6%) (Figure 4, Table 3).

**Table 3:** Biochemical response results

**Follow up time (weeks)**	**Number of studies**	**Test of hetergeneity**		**Analysis model**	**Results**		**RR (95% CI)**	**P-value**
		**I**^**2**^	**P**		**LAM + ADV (n/N)**	**ETV (n/N)**		
12	4	0%	0.43	Fixed effect model	37/102	42/110	0.91[0.65,1.29]	0.61
24	4	37%	0.19	Fixed effect model	69/102	79/110	0.83[0.47,1.49]	0.54
48	4	68%	0.03	Randomed effect model	128/140	120/147	1.09[0.95,1.25]	0.21
96	3	43%	0.17	Fixed effect model	104/108	91/105	1.11[1.02,1.21]	0.01

**Figure 5:**
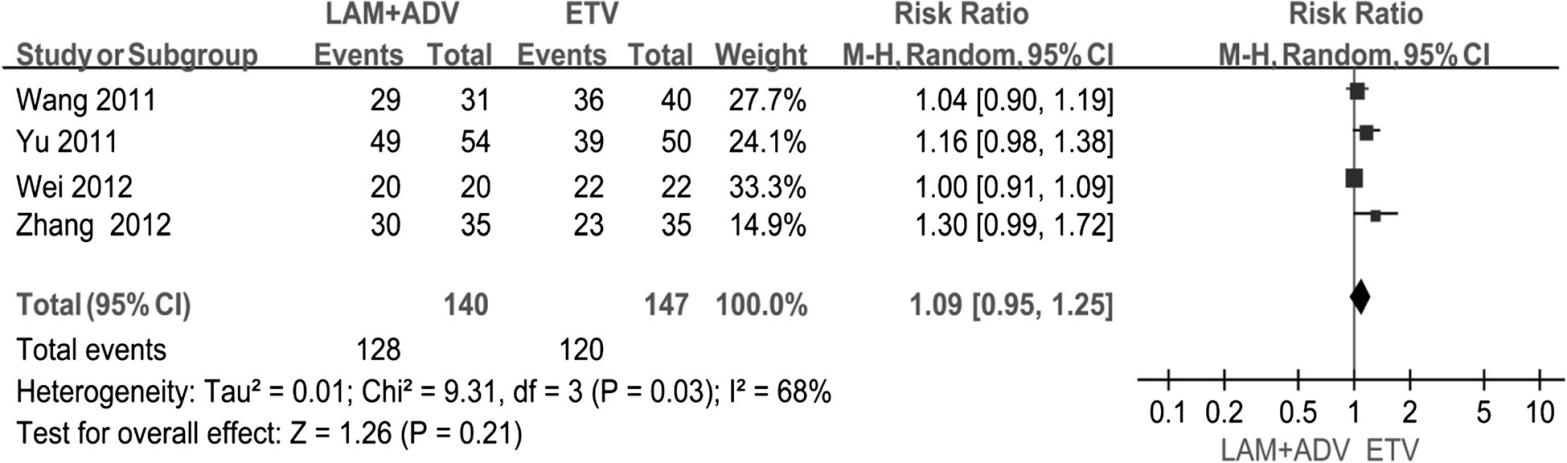
Effect of LAM + ADV vs. ETV at 48 weeks on biochemical response.

There were three studies [22,24,25] that reported the ALT normalization rates at 96 weeks. Chi- and I square (I^2^) analyses showed no heterogeneity [Chi^2^ = 3.51, df = 2, (P = 0.17), I^2^ = 43%]; a summary estimate of the relative risk using a fixed-effect approach was performed. The results showed that the ALT normalization rate in the combination group was superior to ETV group (96.3% vs. 86.7%). The difference between the two groups was statistical significant difference [RR = 1. 11, 95% CI (1.02, 1.21), P =0.01] (Figure 5).

### HBeAg seroconversion

**Figure 6:**
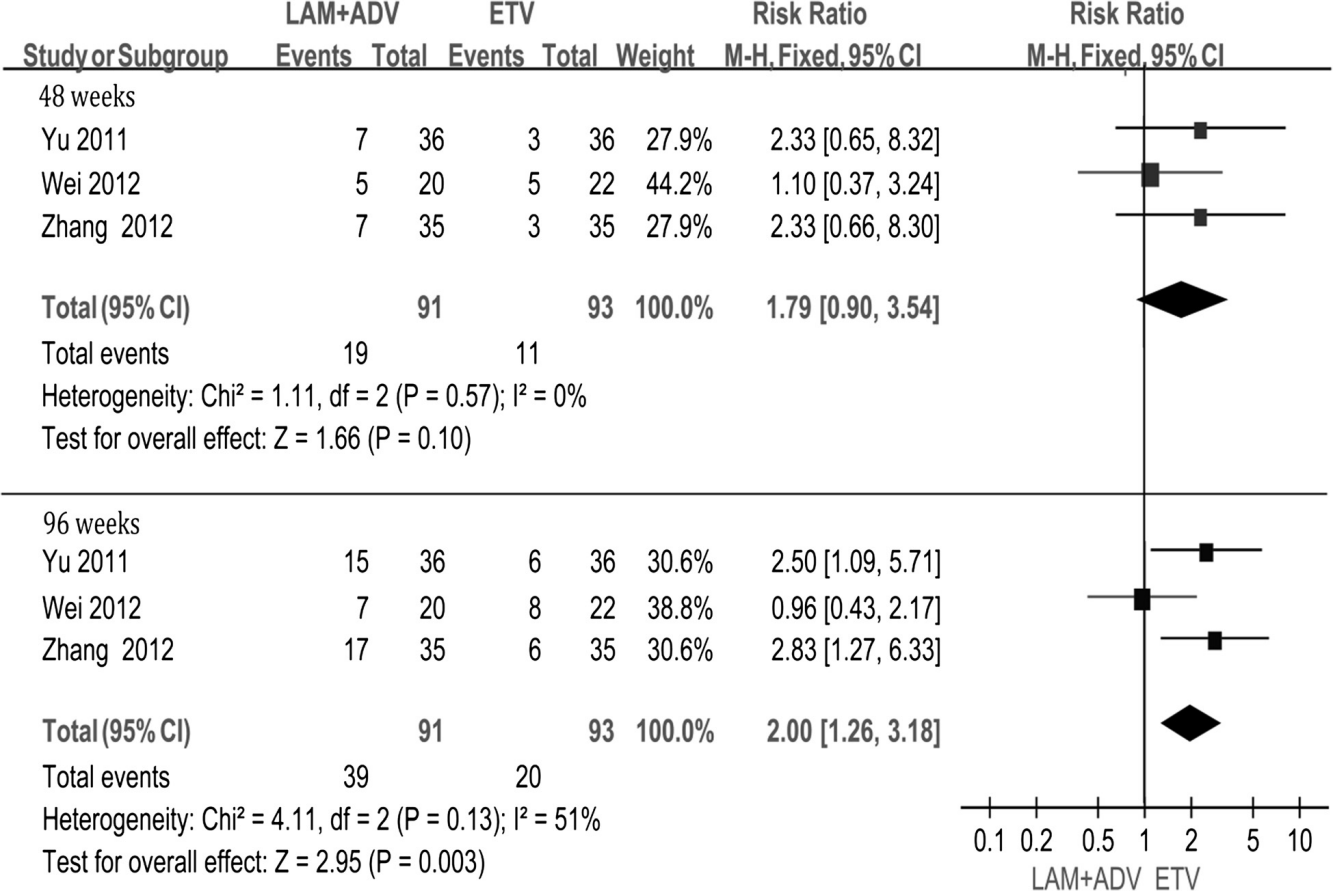
Effect of LAM + ADV vs. ETV on HBeAg seroconversion.

Three studies [22,24,25] provided the data regarding HBeAg seroconversion after 48 and 96 weeks of treatment. Chi- and I square (I^2^) analyses showed no heterogeneity. A fixed-effect analysis showed that the HBeAg seroconversion rate in the LAM + ADV group was distinctly higher than that of in ETV group at both 48 and 96 weeks (20.9% vs. 11.8%, 42.9% vs. 21.5%, respectively). The difference was not statistically significant at week 48 (RR = 1. 79, 95% CI (0.90, 3.54), P =0.10. However, with prolonged duration up to 96 weeks, the difference became statistically significant [RR = 2.00, 95% CI (1.26, 3.18), P =0.003] (Figure 6).

### Viral breakthrough

No viral breakthrough was reported in the combination group. However, six patients experienced viral breakthrough in ETV group. Four patients reported in the study by Yu *et al*. [22] had viral breakthrough. Three had genetic mutations conferring to ETV (two had rtL180M, T184L and M204I mutations; one had rtL180M and M204V LAM-resistance mutations). Further testing on the four patients disclosed that two patients had LAM genotypic resistance mutations (rtL180M and M204V) at the beginning of treatment. Another two patients developed viral breakthrough as mentioned in the Zhang *et al*. [24] study. One patient had ETV resistance mutations (rtL180M + T184L + M204V), and the other had S202G/I and M204V mutations.

### Safety

Wang *et al*. [21] reported that in the combination group, one patient’s the blood urea nitrogen (BUN) increased to 7.87 mol/L at week 24 without a concomitant increase in serum creatinine (Scr). Yu *et al*. [22] reported one patient in the ETV monotherapy group had Scr elevated to 48 mol/L. The abnormal values were remained within normal limits during the period of treatment without receiving any additional treatment or adjustment of dosage. No serious events were reported in either group.

## Discussion

Currently, all NAS approved to treat CHB have inhibitory effects on the HBV polymerase/reverse transcriptase, which has poor proofreading and editing ability, resulting in error-prone replication [26]. Additionally, the high replication rate of HBV, continued existence of the replication template covalently closed circular (cccDNA) and complexity of quasispecies [27,28] increase the likelihood of the development of drug resistance, especially during prolonged therapy. Once viral breakthrough or drug resistance occurs, rescue therapy was been initiated. But, rescue therapy can promote the development of multidrug mutations [19]. Therefore, identification of novel treatment targets remains a major clinical challenge to improve the efficacy of antiviral therapy and prevent drug-resistance.

It has been demonstrated that the main mutations associated with LAM-resistance are rtM204I and/or rtL180M, and these do not confer resistance to ADV [29,30]. The mutations associated with resistance ADV are mainly rtA181V and/or rtN236T [31]. Recently, it has been reported that a single amino acid change at position rt181 may induce cross-resistance to LAM and ADV [32]. However, this phenomenon has not been widely observed in clinical practice. The lack of cross-resistance has provided a rationale for the combination of LAM and ADV therapy, which may be the main reason why viral breakthough in the combination group was not observed in the study. In contrast, six patients in ETV monotherapy developed viral breakthrough and five patients had documented ETV–resistant mutations. Two cases had LAM-resistant mutations before onset of therapy (rtM204I/V ± rtL180M). Previous studies have confirmed that the presence of rtM204I/V and rtl180 M can reduce the genetic resistance barrier to ETV [33,34]. Therefore, pretreatment resistance testing may be useful in detecting viral mutations. If ETV-resistance mutations are present, LAM + ADV combination therapy may be a better choice than ETV in terms of avoiding drug resistence.

The study by Zhang *et al*. [24] included patients with higher baseline HBV DNA level (HBV DNA load >10^7^ copies/mL) than that in the current study. They found that the HBV DNA load decreased to less than 10^3^ copies/mL in 2.9% in the combination group compared with 14.3% in monotherapy group at 12 weeks (P < 0.05). Yu *et al*. [22] also reported that the rate of patients who had HBV DNA load declines to less than 1 copy/ml in was lower in combination group than that in the monotherapy group at 12 weeks (3.7% vs. 18.0%, P = 0.018). In another study, Jayakumar *et al*. [23] found that the median decrease in HBV DNA levels in a combination group were 92.73% and 99.57% at 12 and 24 weeks of therapy, respectively (considering the baseline HBV DNA levels to be 100%). However, in an ETV group, the median decrease was 99.74% and 100%, respectively, in the same follow-up periods. The differences were highly significant at both 12 (P =0.0007) and 24 (P = 0.0115) weeks between the two groups. Furthermore, the results of the analysis prove that LAM plus ADV combination therapy produced more rapid and significant reduction in HBV DNA levels at 12 weeks of therapy, compared with ETV monotherapy, even though there was no significant difference observed at 96 weeks post-treatment. Unfortunately, the raw data were presented in the included studies as percentage of patients with undetectable HBV DNA instead of a decline in level of HBV DNA. Therefore a statistical analysis was not performed.

One potential benefit of rapid suppression of HBV replication is to reduce the risk of drug resistance. It has been shown that the subsequent chance of LAM resistance is directly proportional to the HBV DNA levels after 24 weeks of treatment [35]. A study of LDT versus ADV showed that suppression of HBV DNA levels at 24 weeks correlated with efficacy outcomes and viral breakthrough at 1 year [36]. Longer-term studies with ETV have reported that most patients achieve HBV DNA suppression within the first 24 weeks of treatment, and suppression is maintained for up to 4 years [37] with minimal drug resistance [38]. Thus, the de novo combination LAM and ADV were expected to achieve a better outcome than that in ETV monotherapy in terms of multidrug resistance.

The current review has shown that the combination group had significantly higher rate of HBeAg seroconversion and ALT normalization compared with the ETV group up to 96 weeks. The high rate of HBeAg seroconversion may be related to the rapid reduction of HBV DNA load at week 12 as mentioned above. Other studies have verified that an early reduction of HBV DNA is associated with an increased likelihood of HBeAg seroconversion [39]. He *et al*. [40] reported that at 24 weeks, the rates of HBV DNA negativity in the LAM and ADV combination group were higher than that in the LAM or ADV monotherapy group. At week 96, the percentages of patients with undetectable DNA and HBe seroconversion in the combination group (100%, and 51%, respectively) were superior to LAM (66%, 21%, respectively) or ADV alone groups (49%, 33%, respectively). Ghany *et al*. [3] also found that combination therapy with LAM and ADV was associated with a high rate of virological and biochemical response both at week 48 (59% vs. 26%, P = 0.06). At week 192, 76% of the combination vs. 36% of the monotherapy groups had loss of HBeAg (P = 0.03). The current treatment guidelines recommend that antiviral therapy be stopped for HBeAg-positive CHB patients (except those with cirrhosis) when either HBeAg is undetectable or when HBeAg seroconversion has taken place, and when HBV DNA is undetectable for an additional 6-12 months after continuous therapy [8]. HBeAg loss or seroconversion may reduce the risk of developing HCC or progressive liver diseases [41] in HBeAg-positive CHB patients. This suggests that superiority of combination of LAM and ADV over ETV alone in term of HBeAg seroconversion may provide potential benefits in patients with HBeAg-positive CHB.

The potential for an increased risk of toxicity must always be noted when instituting combination LAM and ADV. In this meta-analysis, either combination group or ETV alone was well tolerated. Only one patient presented with an elevated BUN in the combination group. The levels were monitored closely and remained within normal limits during the treatment period. With prolonged durations of the combination treatment, not only effects, but also costs should be evaluated. Wu et al. [42] observed that the initiation of rescue therapies for LAM-resistant CHB with adding ADV is likely to be more cost-effective than ADV, ETV, TDF monotherapy. In the current study, a cost-effectiveness analysis was not done because costs of medications were not included.

The limitations of this review include the fact that some studies were not RCTs. Publication bias was also possible. Compared to positive studies, negative studies may be less likely to be published and more likely to take longer to be published. This could have affected the validity of the meta-analysis in this review [43]. In addition, many studies were conducted in China resulting in regional and language bias. Finally, there were only a few studies and the sample size was small.

In conclusion, de novo combination of LAM and ADV therapy for naïve treated patients was not superior to the ETV monotherapy in short duration; however, the combination therapy had higher biochemical response and HBeAg seroconversion rates compared with monotherapy when the therapy duration was prolonged up to 96 weeks. The rate of emergence of viral resistance in combination group was less than that in the ETV group. However, given the limited number of studies included in the analysis, caution should be exercised in extrapolation of the conclusion to all patients infected with CHB. More high-quality, well-designed, randomized controlled, multicenter studies are clearly needed to confirm these observations.

## Abbreviations

ALT: Alanine aminotransferase; BUN: Blood urea nitrogen; cccDNA: Covalently closed circular DNA; CHB: Chronic Hepatitis B; CI: Confidence Intervals; HBV: Hepatitis B virus; HCC: Hepatocellular Carcinoma; HIV: Human immunodeficiency virus; RCTs: Randomized controlled trials; NAS: Nucleos(t)ide analogues; Scr: Serum creatinine(Scr).

## Competing interests

The authors declare that they have no competing interests.

## Authors’ contributions

HP and RH conceived the study, provided fund supporting and revised the manuscript critically for important content. LF made substantial contributions to its design, acquisition, analysis and interpretation of data. WF, WXW, HHD and ZDZ participated in the analysis and interpretation of data. All authors read and approved the final manuscript.

## Authors’ information

Fen Liu is a master’s degree candidate with a major of infectious disease medicine in The Second Affiliated Hospital of Chongqing Medical University, Chongqing, Chongqing, China.
